# Lipidomics Analysis of the Tears in the Patients Receiving LASIK, FS-LASIK, or SBK Surgery

**DOI:** 10.3389/fmed.2021.731462

**Published:** 2021-10-27

**Authors:** Yan Gao, Yuanyuan Qi, Yue Huang, Xiaorong Li, Lei Zhou, Shaozhen Zhao

**Affiliations:** ^1^Ocular Proteomics Platform, Singapore Eye Research Institute, Singapore, Singapore; ^2^Tianjin Key Laboratory of Retinal Functions and Diseases, Tianjin Branch of National Clinical Research Center for Ocular Disease, Eye Institute and School of Optometry, Tianjin Medical University Eye Hospital, Tianjin, China; ^3^Department of Ophthalmology, Yong Loo Lin School of Medicine, National University of Singapore, Singapore, Singapore; ^4^Ophthalmology and Visual Sciences Academia Clinical Program, Duke-National University of Singapore Medical School, Singapore, Singapore

**Keywords:** lipidomics, refractive surgery, LASIK, FS-LASIK, SBK, PLS-DA

## Abstract

**Purpose:** Tear film lipid layer (TFLL) plays a vital role in maintaining the tear film stability and, thus, the lipid composition of the tears could greatly affect the physiological function and biophysical integrity of the tear film. The objective of this study is to assess the tear lipid composition of the patients receiving laser-assisted *in situ* keratomileusis (LASIK), femtosecond LASIK (FS-LASIK), or sub-Bowman's keratomileusis (SBK) surgery preoperatively and postoperatively.

**Methods:** Tear samples were collected from the left eye of the patient who receiving LASIK (*n* = 10), FS-LASIK (*n* = 10), or SBK (*n* = 10) surgery in week 0, week 1, week 4, and week 52. A rapid direct injection shotgun lipidomics workflow, MS/MS^ALL^ (<2 min/sample), was applied to examine the tear lipidome.

**Results:** In week 52, the SBK group demonstrated a similar lipidome profile compared to week 0, while the FS-LASIK and LASIK groups shifted away from week 0. Two lipids, ganglioside (GD3) 27:4 and triacylglycerol (TAG) 59:3, were found to be associated with the lipidome changes preoperatively and postoperatively. No statistical significance was found in the overall lipid classes from the FS-LASIK group. The LASIK group showed significant alteration in the phospholipid and sphingolipid over time, while the SBK group demonstrated a significant difference in the (O-acyl)-ω-hydroxy fatty acid (OAHFA) and phospholipid.

**Conclusion:** LASIK showed the greatest impact on the tear lipidome changes over time, while SBK demonstrated minimal impact among the three types of refractive surgeries after 1 year.

## Introduction

The thin layer of the tear film covers the anterior surface of the cornea and serves the critical functions in maintaining the proper ocular function and health. Its main roles include moistening the mucous membrane, nourishing the avascular corneal, flushing out the contaminants and irritants, and providing a smooth surface for the visual acuity (1, 2). It has been proposed that the tear film is composed of three layers: an inner mucin layer, a middle aqueous layer, and an outer tear film lipid layer (TFLL). Lam et al. further divided the lipid layer into two sublayers: the superficial sublayer mainly consisting of the non-polar lipids and an inner amphiphilic sublayer facilitating the interaction between the polar and non-polar components of the tears (3). TFLL is vital for a stable tear film by preventing the tear film from evaporation (4). Therefore, the physiological function and biophysical integrity of the tear film would be greatly affected by the lipid composition. It was a challenging task to fully evaluate the lipid profile of the tear samples considering the small amount of the materials obtained from the humans, the diversity of the lipid species, and the complexities of the qualitative and quantitative lipidomics analysis (5). Nevertheless, the lipid composition of the tear film has been extensively studied (6–8). The high sensitivity of the mass spectrometry (MS) in analyzing the low sample volumes makes it a preferred approach in the biomedical research to decipher the fine changes of the lipid metabolism in the ocular and nonocular disorders, for example, Meibomian gland dysfunction, dry eye syndrome (9), and multiple sclerosis (10).

There are two techniques used for the excimer laser refractive correction procedures: surface or stromal ablation. The shallow cornea disruption in the surface ablation procedures such as photorefractive keratectomy (PRK), laser epithelial keratomileusis (LASEK), and epithelial laser-assisted *in situ* keratomileusis (Epi-LASIK) results in a lower incidence of the surgery-induced dry eye and provides more stability for the thinner cornea, implicating the better biomechanical outcomes. However, greater discomfort caused by the wound response and delayed vision recovery would still be the major problems for the surface ablation technique (11, 12). In contrast, the stromal ablation surgeries such as LASIK, femtosecond LASIK (FS-LASIK), and sub-Bowman's keratomileusis (SBK) have the advantages of essentially immediate vision correction, quick recovery, and very little to no discomfort (13). These three types of the refractive surgery use the corneal flap creation procedure to maintain the integrity of the corneal structures such as the Bowman's layer and the epithelium (14). However, the risk of post-LASIK keratectasia was elevated for the patients with moderate to high myopia due to the thicker flap (110–160 μm) (15, 16). SBK was developed from LASIK by using a mechanical microkeratome to create a thinner corneal flap (90–110 μm) and more planar in shape compared with the conventional LASIK approach (17). This approach is an evolutive procedure that increased the biomechanical stability of LASIK and reduced the pain experience of PRK (18, 19). The variation of the flap thicknesses and flap diameters by using a mechanical microkeratome in LASIK and SBK approaches was still a problem, despite their safeness and effectiveness. Corneal flap creation has become a more predictable and safe procedure with the introduction of FS-LASIK (20, 21).

Dry eye is a common symptom after LASIK surgery. It is believed that postsurgical development of the dry eye is closely related to the surgical cut of the corneal nerve fibers during the flap creation (22) and associated with the degree of preoperative myopia and the depth of laser treatment (23). The loss of the corneal innervation could affect the lacrimal function unit (LFU) (24), corneal blinking, and blinking of the Meibomian gland reflexes, resulting in the decreased aqueous and lipid tear secretion and mucin expression (25). Patel et al. showed that the tear lipid layer became thinner after LASIK (26). In general, patients receiving FS-LASIK surgery demonstrated the stable tear film compared with the mechanical microkeratome group (27, 28).

Although the tear lipids have been widely studied, a comprehensive lipidomics study examining the tear lipid profiles before and after the refractive surgery is still lacking. In this study, a technique specifically designed for the global lipidomics, MS/MS^ALL^, was used to assess the tear lipidome prior to and after the refractive surgery (29–31). This technique collects all the precursor ions in the Q1 quadrupole and the collision-induced dissociation is carried out in Q2 quadrupole while collecting all the high-resolution MS/MS spectra at a high speed (29, 30). MS/MS^ALL^ is highly reproducible, bias free, and requires no method development (32). Given the important role of TFLL in maintaining the proper ocular function and easy accessibility of the tear samples, the tear lipid compositions of the patients taken FS-LASIK, LASIK, or SBK surgery preoperatively (week 0) and postoperatively (week 1, week 4, and week 52) were investigated by using MS/MS^ALL^ in this study.

## Methods and Materials

### Sample Collection

The study was approved by the Tianjin Medical University Institutional Review Board and was conducted according to the Declaration of Helsinki. The signed consent forms were obtained from the participating volunteers. The criteria for this study include: (1) the age of the participants should be over 18 years old; (2) a stable refractive error in the last 1 year; (3) soft contact lenses had not been worn for more than 1 week; (4) rigid contact lenses had not been worn for more than 2 weeks; (5) no history of eye disease or eye surgery; (6) no systemic connective tissue disease or autoimmune disease; (7) no other systemic diseases (such as diabetes, seborrheic dermatitis, or hyperlipidemia); (8) postoperative corneal stromal bed thickness was >250 μm; and (9) no breastfeeding or pregnancy. All the patients were explained about the advantages, disadvantages, and the risk of the three types of surgeries. The type of surgery to be carried out in each patient was based on the preference of the patient. Clinical examinations for the patients receiving FS-LASIK (*n* = 10), LASIK (*n* = 10), or SBK (*n* = 10) surgery included Schirmer test (without anesthesia), tear breakup time (TBUT), and corneal fluorescein staining. Corneal staining was graded from 0 to 5 according to the Oxford schema (33). Tear samples were collected by using the Schirmer strips from both the eyes of the patients and the strips were stored at −80°C until further analysis. Postregime for all the patients is the same. Topical medications after surgery consisted of fluorometholone eye drops four times daily for 1 week and tapered over 4 weeks, levofloxacin eye drops three times per day for 3 days, and artificial tears four times daily for 1–3 months depending on the severity of the postsurgical dry eye symptoms.

A metal spatula was used to collect the expressed meibum, which was generated by gentle squeezing the eyelids of the volunteers. Meibum lipids were eluted by washing the spatula thoroughly with chloroform and the lipid extracts were subjected to dry by using a miVac sample concentrator (Genevac, Ipswich, UK). The dried samples were stored at −80°C until further analysis.

### Lipid Extraction

The protocol for the lipid extraction was adopted from the previous work with some modifications (34). The first 15 mm of Schirmer strips were cut into the fine pieces (~2 mm) in the glass tubes. About 200 μL of methanol containing 50 μg/ml butylated hydroxytoluene (BHT) and 25 ng/ml myristic-d27 acid were added to the glass tube followed by 600 μL methyl tert-butyl ester (MTBE). The mixture was then incubated at 20°C for 30 min with a mixing speed of 900 rpm. About 180 μL of water was added for the phase separation. After thoroughly mixing the sample, the suspension is centrifuged for 10 min at 10°C with a speed of 2,000 *g*. The upper phase containing lipid was then transferred into a collection vial and dried down.

### Direct Injection MS/MS^ALL^ Data Acquisition

Lipid extract was reconstituted in 100 μL methanol/chloroform (2:1, v/v) with 5 mM ammonium acetate and the sample was automatically loaded and directly delivered to the electrospray ionization (ESI) source by using the ACQUITY UPLC I-Class System (Waters Corporation, Milford, Massachusetts, USA). The running buffer was methanol/isopropanol (3:1, v/v) with 5 mM ammonium acetate and the flow rate was 30 μL/min. The MS/MS^ALL^ acquisition experiment was carried out on the SCIEX TripleTOF 5600 System (SCIEX, Framingham, Massachusetts, USA) in both the positive and negative polarities for the complete lipidome coverage. The parameter settings for ESI source included nebulizing gases (GS1) at 25, heating gases (GS2) at 10, curtain gas (CUR) at 20, temperature at 250°C, and ion spray voltage floating at 5,500 V for positive ionization and −4,500 V for negative ionization, respectively. The atmospheric pressure chemical ionization (APCI) probe and inlet were connected to an external calibrant delivery system (CDS) delivering the mass calibration solution for MS and MS/MS. The Analyst® TF 1.7 software (SCIEX, Framingham, Massachusetts, USA) was used to acquire the data from MS/MS^ALL^. The mass range for time-of-flight MS (TOFMS) was from 200 to 1,200 m/z and the accumulation time was 300 ms, followed by 1,000 MS/MS spectra from 200.050 to 1,200.049 m/z in 1 Da steps. The accumulation time for the product ion scan was 100 ms and the collision energy was set to 50 ± 30 eV for positive polarity and −45 ± 30 eV for negative polarity, respectively. The total run time for one MS/MS^ALL^ acquisition was <2 min.

### Raw Data Processing

The lipid identification and quantitation were performed by using the LipidView™ software 1.2 (SCIEX, Framingham, Massachusetts, USA) with a built-in library containing glycerolipids, phospholipids, sphingolipids, sterol lipids, and fatty acyls. A targeted search list for the wax esters was also included. A background subtraction by using the Schirmer strip and solvent was applied to the sample.

### Statistical Analysis

The results are expressed as mean ± SD. Clinical characteristics were compared among LASIK, FS-LASIK, and SBK participants by the chi-squared test, a one-way ANOVA, or the Kruskal–Wallis one-way ANOVA as appropriate. The analysis of the lipids over the course of time was conducted by using the ANOVA by R programming (35). The principal component analysis (PCA) and partial least squares-discriminant analysis (PLS-DA) were carried out by the MetaboAnalyst 4.0 (Xia Lab@McGill, Quebec, Canada) (36) and the SIMCA 13.0.3 (Umetrics, Sweden, UK).

## Results

### Clinical Characteristics of the Patients

**Figure 1:**
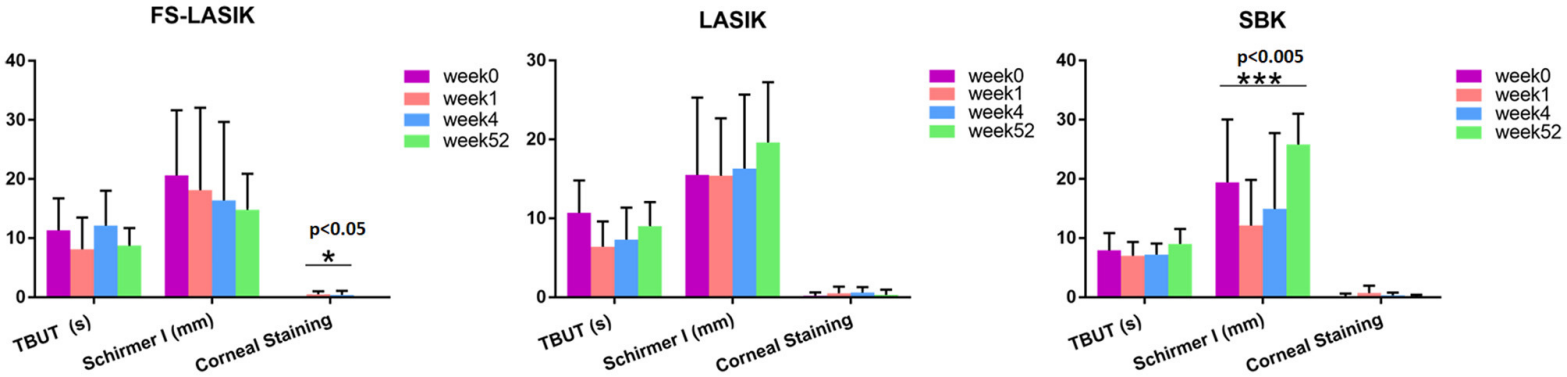
Clinical examination of the patients receiving femtosecond laser-assisted *in situ* keratomileusis (FS-LASIK), LASIK, and sub-Bowman's keratomileusis (SBK) surgery in week 0, week 1, week 4, and week 52, respectively. ^*^p < 0.05, ^**^p < 0.01, ^***^p < 0.005.

Table 1 shows the characteristics of the recruited patients in this study. There is no significant difference in age, gender, spherical, cylindrical, TBUT, Schirmer test, or corneal staining results among LASIK, FS-LASIK and SBK group prior to the refractive surgery. The assessment of TBUT, Schirmer test, and corneal staining for the patients receiving FS-LASIK, LASIK, or SBK surgery in week 0, week 1, week 4, and week 52 was shown in Figure 1. A significant difference in the corneal staining was noted in the FS-LASIK group over time. There was also a significant difference in the Schirmer test for the SBK group.

**Table 1:** Characteristics of the patients receiving FS-LASIK, LASIK, or SBK surgery.

	**FS-LASIK**	**LASIK**	**SBK**	***p*** **-value**
Patients, *n*	10	10	10	-
Age, years, mean ± SD	21 ± 3	25 ± 5	23 ± 5	0.311
Sex, male, *n* (%)	5 (50%)	5 (50%)	5 (50%)	1.00
**Refractive status**				
Spherical (mean ± SD)	−4.58 ± 1.65	−4.43 ± 1.39	−3.53 ± 1.80	0.311
Cylindrical (mean ± SD)	−0.80 ± 0.40	−1.25 ± 0.53	−1.03 ± 1.39	0.142
**Clinical examinations**				
TBUT (s)^a^	11.30 ± 5.44	10.70 ± 4.11	7.90 ± 2.96	0.186
Schirmer I (mm)	20.60 ± 11.06	15.50 ± 9.82	19.40 ± 10.65	0.534
Corneal staining	0.0 (0.0)	0.0 (0.25)	0.0 (0.25)	0.328

a *FS-LASIK, femtosecond laser-assisted in situ keratomileusis; SBK, sub-Bowman's keratomileusis; TBUT, tear breakup time*.

### Lipid Detection by Using MS/MS^ALL^

To demonstrate the reasonable coverage of the lipid species detected by this ultra-fast MS/MS^ALL^ method, several normal human tears and the meibum samples were evaluated. In this study, a list of the lipid species detected in the tears or meibum by using MS/MS^ALL^ technique was compared with the previous publications (3, 5, 37–41) and the result was shown in Table 2 indicating that this technique is capable of detecting the major lipid classes. We also found 76 new lipid species by using MS/MS^ALL^ method including (O-acyl)-ω-hydroxy fatty acids (OAHFAs), wax ester (WE), triacylglycerol (TAG), diacylglycerol (DAG), phosphatidylcholine (PC), phosphatidic acid (PA), phosphatidylserine (PS), phosphatidylglycerol (PG), lysophosphatidylcholine (LPC), sphingomyelin (SM), monosialodihexosylganglioside (GM3), and cholesteryl ester (CE) (Table 3). In total, around 300 lipid species that are present in ≥75% of the samples were detected in this study.

**Table 2:** Summary of the lipids detected in the tears or meibum by using MS/MS^ALL^.

**Lipid group**	**Lipid class^a^**	**Ionization polarity**	**Number of species**		
			**Reported in tears/** **meibum^b^**	**Tears**	**Meibum**
Fatty acyls	OAHFA	Negative	74	10	33
	WE	Positive	65	44	67
Glycerolipids	TAG	Positive	44	17	0
Glycerophospholipids	PE	Negative	45	0	1
	PI	Negative	18	0	1
	PC	Positive	51	5	0
	LPC	Positive	13	10	0
Sphingolipids	SM	Positive	23	9	0
Sterol lipids	CE	Positive	56	30	40

a *OAHFA, (O-acyl)-ω-hydroxy fatty acid; WE, wax ester; TAG, triacylglycerol; PE, phosphatidylethanolamine; PI, phosphatidylinositol; PC, phosphatidylcholine; LPC, lysophosphatidylcholine; SM, sphingomyelin; CE, cholesteryl ester; MS, mass spectrometry*.

b *Based on work by Butovich (5), Butovich et al. (37), Hancock et al. (39), Chen et al. (38, 40), Rantamäki et al. (41), and Lam et al. (3) groups*.

**Table 3:** New lipid species detected by using MS/MS^ALL^ workflow.

**Lipid group**	**Lipid class^a^**	**Ionization polarity**	**Number of species newly detected in tears/meibum (# = 76)**
Fatty acyls	OAHFA	Negative	13
	WE	Positive	14
Glycerolipids	TAG	Positive	9
	DAG	Positive	1
Glycerophospholipids	PE	Negative	0
	PI	Negative	0
	PC	Positive/Negative	3
	PA	Negative	11
	PS	Negative	5
	PG	Negative	4
	LPC	Positive	7
Sphingolipids	SM	Positive	1
	GM3	Positive	5
Sterol lipids	CE	Positive	3

a *OAHFA, (O-acyl)-ω-hydroxy fatty acid; WE, wax ester; TAG, triacylglycerol; DAG, diacylglycerol; PE, phosphatidylethanolamine; PI, phosphatidylinositol; PC, phosphatidylcholine; PA, phosphatidic acid; PS, phosphatidylserine; PG, phosphatidylglycerol; LPC, lysophosphatidylcholine; SM, sphingomyelin; GM3, monosialodihexosylganglioside; CE, cholesteryl ester*.

### Multivariate Analysis of the Lipidomic Profile

**Figure 2:**
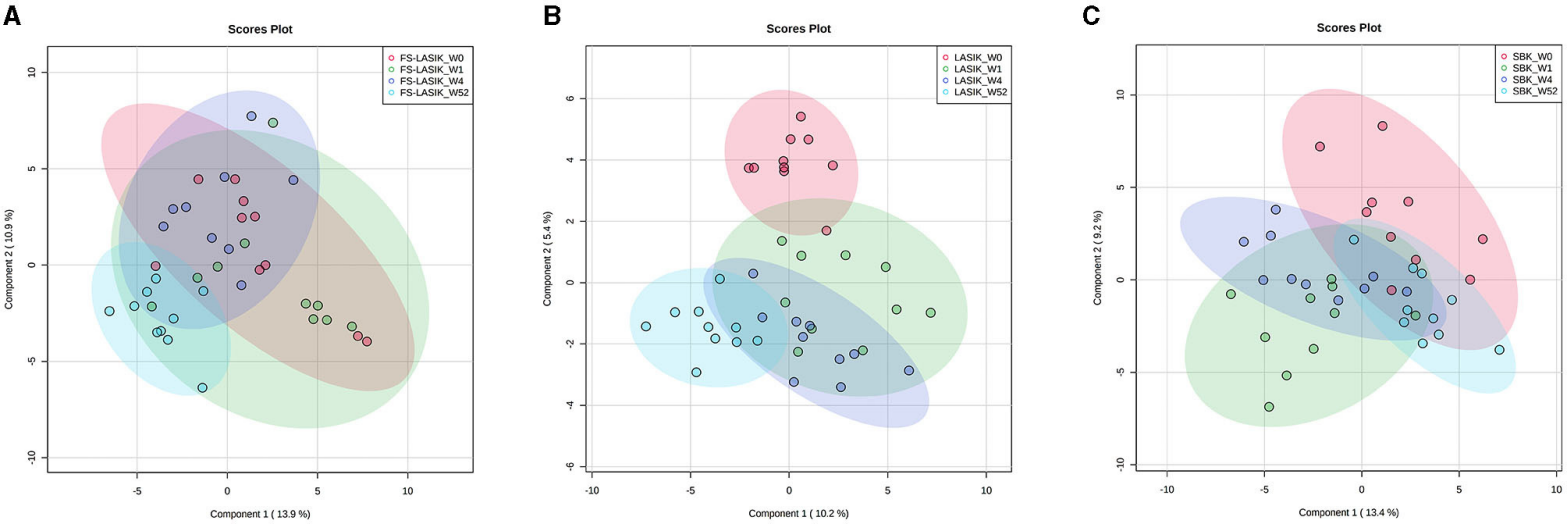
The partial least squares-discriminant analysis (PLS-DA) score plots showing the lipid profiles in week 0, week 1, week 4, and week 52 from the FS-LASIK, LASIK, and SBK groups, respectively. **(A)** FS-LASIK; **(B)** LASIK; **(C)** SBK. Red circle: week 0; Green circle: week 1; Purple circle: week 4; and Blue circle: week 52.

Multivariate analysis including PCA and PLS-DA was applied in this study to examine the pattern of the lipidomic profiles over time (week 0, week 1, week 4, and week 52). The tight cluster of the quality control (QC) samples in PCA score plot indicated the robustness of our direct injection MS/MS^ALL^ data acquisition platform (Supplementary Figure 1). An overview of the lipidomic profiles preoperatively and postoperatively from the FS-LASIK, LASIK, and SBK groups was shown in Figure 2 by using the PLS-DA score plots. There was ample overlap among week 0, week 1, and week 4 in the FS-LASIK group, implicating there was no clear difference among these three time points. In contrast, the lipidomic profiles of the LASIK and SBK groups in week 1 and 4 were distinctly separated compared to week 0. Interestingly, an overlap between week 0 and 52 was observed in the SBK group. On the other hand, the LASIK group showed a larger difference compared to the FS-LASIK group between week 0 and 52. In addition, the first two components of the PLS-DA model for the three types of surgeries can only explain ~20% of covariance among the different time points. Individual variations and small sample size might be one reason for this covariance. The above findings revealed that in week 52, the SBK group demonstrated a similar lipidome profile compared to week 0, while the FS-LASIK and LASIK groups shifted away from week 0.

**Figure 3:**
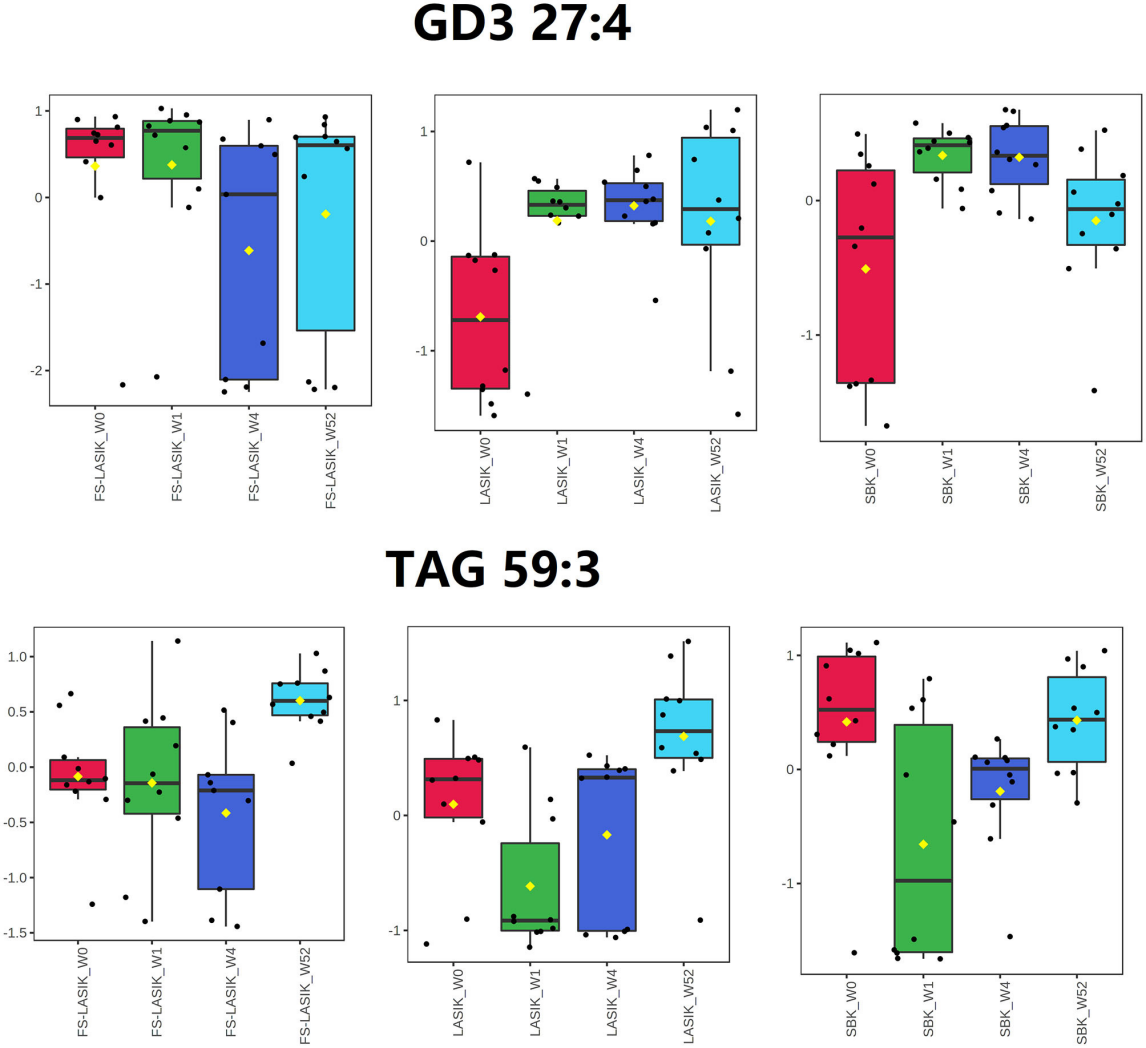
Box and whisker plots showing the intensity changes over time for GD3 27:4 and triacylglycerol (TAG) 59:3.

The loading plot of the PLS-DA model is complementary to the score plot and summarizes how the lipids relate to each subgroup. By examining the corresponding loading plot of the PLS-DA model (Supplementary Figure 2), TAG 59:3 is closely associated with the FS-LASIK and LASIK groups in week 52. In addition, GD3 27:4, a disialoganglioside with the three glycosyl groups, is closely associated with the FS-LASIK and SBK groups in week 1. Interestingly, this lipid is associated with the lipidomics profile of the LASIK group in week 4. The levels of these two lipids in the FS-LASIK, LASIK, and SBK groups preoperatively and postoperatively were shown in Figure 3.

### Comparison of the Lipid Classes and Lipid Species Among the Refractive Surgeries

**Figure 4:**
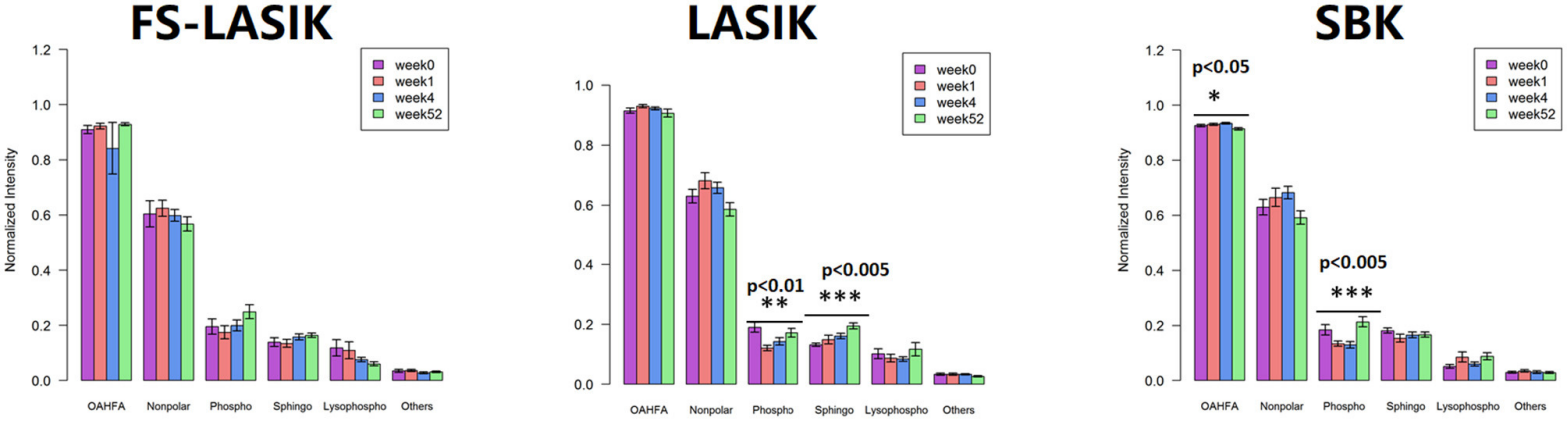
Overall tear lipid classes at week 0, week 1, week 4, and week 52 from the FS-LASIK, LASIK, and SBK groups. ^*^p < 0.05, ^**^p < 0.01, ^***^p < 0.005.

The levels of the major lipid classes found in the FS-LASIK, LASIK, and SBK groups in week 0, week 1, week 4, and week 52 were shown in Figure 4. In this study, the major lipid classes include OAHFAs, non-polar lipid, phospholipid, sphingolipid, and lysophospholipid. No statistical significance was found in the overall lipid classes from the FS-LASIK group. The LASIK group showed significant alteration in the phospholipid and sphingolipid over time, while the SBK group demonstrated a significant difference in the OAHFA and phospholipid.

**Figure 5:**
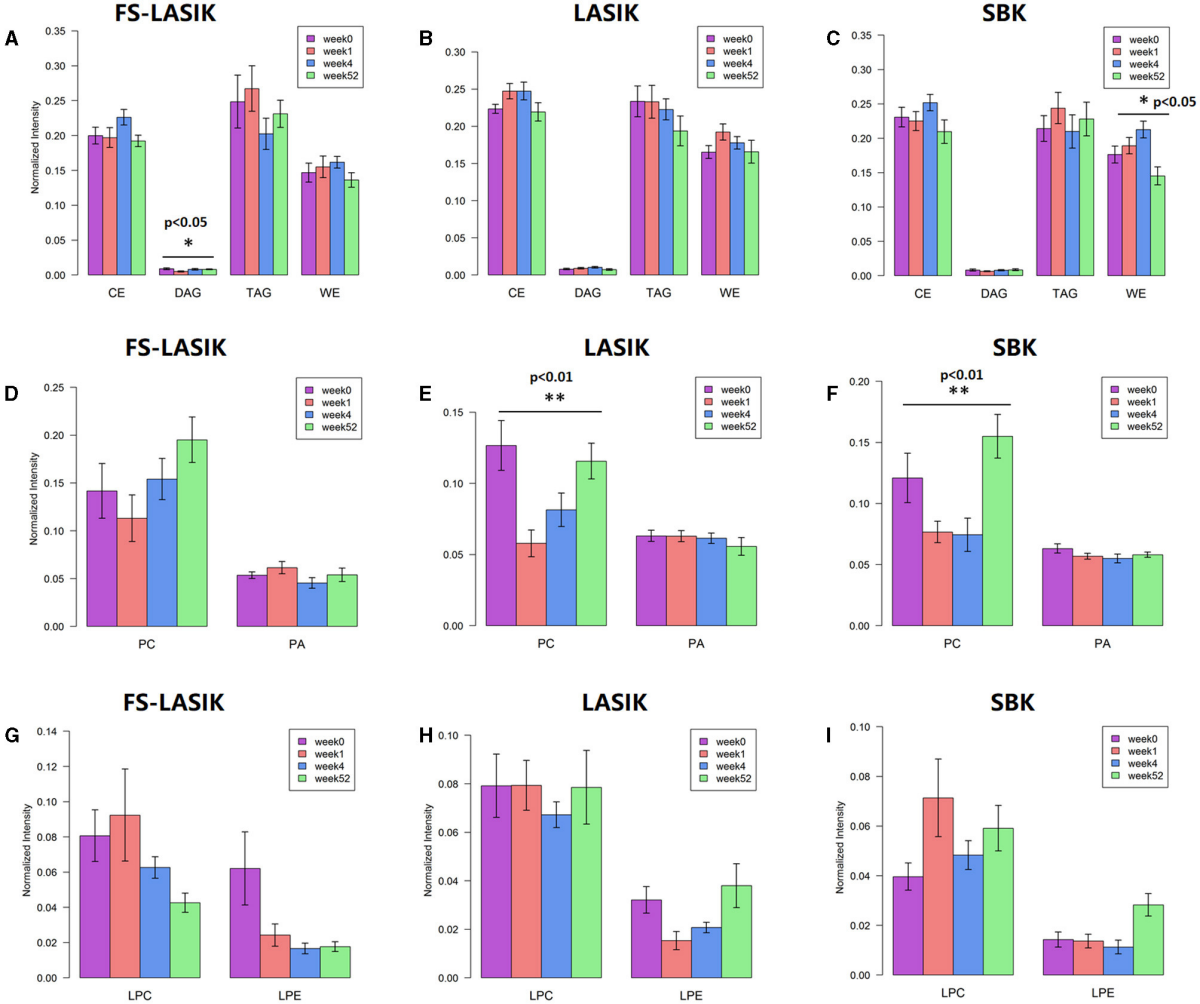
Individual lipid classes found in the FS-LASIK, LASIK, and SBK groups at week 0, week 1, week 4, and week 52, respectively. **(A–C)** Non-polar lipid; **(D–F)** phospholipid; **(G–I)** lysophospholipid. ^*^p < 0.05, ^**^p < 0.01, ^***^p < 0.005.

Individual lipid species belonging to non-polar lipid, phospholipid, and lysophospholipid in week 0, week 1, week 4, and week 52 were also examined in this study and the quantitative comparison from the FS-LASIK, LASIK, and SBK groups was shown in Figure 5. The levels of DAG in the FS-LASIK and WE in the SBK groups were significantly changed over time (Figures 5A,C). Most of the difference in the phospholipids was observed in PC from the LASIK and SBK groups with statistical significance. No significant change was detected in lysophospholipid.

## Discussion

Tear samples have gained popularity in the investigation of disease pathogenesis (1), progression (7), and treatment response (42) due to its quick and non-invasive collection. The content of tear is a dynamic reflection of the ocular surface and, therefore, the lipidomic profiling analysis of the tear could provide information of the physiological, nutritional, and health status of an individual. A high-resolution MS/MS^ALL^ shotgun lipidomics analysis was applied here to investigate the tear samples from the patients receiving LASIK, FS-LASIK, or SBK surgery preoperatively and postoperatively.

In this study, direct injection-based MS shotgun lipidomics combined with liquid pump and autosampler from liquid chromatography (LC) with MS to perform the rapid lipidomic profiling. Comprehensive profiling and quantitation of lipid species could be achieved by this approach without the front-end chromatography separation (43). It captured every precursor ion by high-resolution MS/MS without missing any information. Therefore, MS/MS^ALL^ could obtain quantification information with no method development required for all the species in a single analysis. The use of a fully automated sampler and short run time (around 2 min for each sample) makes high-throughput sample analysis applicable for future clinical applications. Lastly, this approach allows for the detection of most lipid species reported in the literature and some new lipid species, implicating the feasibility of the direct injection based-MS shotgun lipidomics.

Dry eye after the stromal ablation surgeries is closely related to the corneal denervation. The stromal and sub-basal nerves are both severed during the flap creation, except those located at the flap hinge. As a consequence of the severed nerves, a reduction in the tear film stability and dry eye symptoms may occur (44). In this study, we only detected the significant changes in the corneal staining that results in the FS-LASIK group and the Schirmer test in the SBK group. Dry eye after the laser corneal refractive surgery is considered the most common complication with clinical signs such as positive vital staining of the ocular surface, decreased TBUT and Schirmer test value, reduced corneal sensitivity, and decreased functional visual acuity (45). However, previous literature is inconsistent with respect to the tear film stability after the refractive surgery. Some have reported that TBUT and Schirmer test value were diminished in both the microkeratome and femtosecond laser-created flaps (46–49), while others found no significant changes in TBUT and Schirmer test or noted a slight but insignificant increase in TBUT (50–53). There is also a discrepancy in the corneal staining that results after the refractive surgery. Some research groups observed the elevated corneal staining after 1 week of the refractive surgery and it recovered to the baseline levels after 1 month (54, 55). In contrast, Bower et al. reported significantly higher cornea staining for up to 12 months (56). In this study, corneal staining was elevated in week 1 and almost returned to the preoperative levels in week 52 among all the three types of refractive surgery. The sub-basal nerves left in the flap will undergo a degenerative process other than a sudden vanishing after surgery (57, 58). Furthermore, Wilson suggested that the punctate epithelial erosions after surgery may be attributed to the neurotrophic epitheliopathy (50). The creation of the flap by using the microkeratome was irregular and thick compared with the femtosecond laser (59) and, thus, more sub-basal nerves were disrupted and undergoing regeneration after the microkeratome application. The total number of the sub-basal nerve was reported to be negatively correlated with corneal staining (60). It could be a possible reason for the significant changes of the corneal staining overtime in the FS-LASIK group. Another factor to be considered for this observation is the difference in suction time. A femtosecond laser had a longer suction time (~56 s) compared to laser (~40 s) and microkeratome (~20 s) (61). Therefore, the cells of the ocular surface including conjunctival goblet cells may have an increased risk of damage in the FS-LASIK group. In addition, the energy attenuation property of the femtosecond laser during the flap creation process may result in the incomplete dissociation of the corneal flap margin. Lastly, small sample size and individual variation may also affect the clinical observations in this study.

The SBK group showed higher TBUT and Schirmer test value in week 52 compared with preoperation, while the FS-LASIK and LASIK groups had lower TBUT in week 52, implicating the recovery of the SBK group in week 52. This finding is consistent with the PLS-DA score plot results, which demonstrated a similar lipidome profile in week 52 and 0 for the SBK group. The accordance between the clinical examination and lipidomics results indicated that the occurrence and development of dry eye after the refractive surgery are closely related to the lipidome alteration. The creation of the corneal flap is the most critical element in LASIK surgery. FS-LASIK performed the corneal flap creation by using the femtosecond laser, while LASIK and SBK used a mechanical microkeratome. SBK can create a thinner corneal flap compared to LASIK and, hence, increase the available residual stromal bed, preserve corneal tissue, and reduce stromal nerve damage (62, 63). It has been reported that the femtosecond laser could directly trigger the apoptosis of the keratocytes and the corneal flap must be separated bluntly after the application of the femtosecond laser (64). This additional mechanical flap dissection might induce an extra injury for the corneal nerve or tissue cell and, thus, affect the recovery rate. The different patterns of lipidome among the three types of surgeries might also be due to the various tissue reactions caused by the laser or microkeratome application. Zhang et al. found that the corneal sub-basal nerve fibers repairing in the SBK group were faster compared to the FS-LASIK group (17). In addition, the anatomy results of the corneal nerve showed that SBK surgery could conserve more nerve branches compared to FS-LASIK surgery. This study indicates that the thinner corneal flap creation might accelerate the recovery of the lipidomic profile to preoperation after 1 year compared to the lipidome of the SBK group to the FS-LASIK and LASIK groups in week 0 and 52, respectively (Figure 2).

In this study, GD3 27:4 was highly expressed in week 1 compared to week 0 in the LASIK and SBK groups, while the difference between week 0 and 1 in the FS-LASIK group was not obvious. This finding was in accordance with the PLS-DA score plot results that showing the different patterns between week 0 and 1 in the FS-LASIK, LASIK, and SBK groups, respectively. Gangliosides are a family of acidic glycosphingolipids and GD3 is a minor ganglioside in most normal tissue. Gangliosides might play a role in the biological processes related to the retinal physiology and vision disorders involving the loss of photoreceptors or pathological retinal neovascularization (65). The expression of GD3 ganglioside increases during the development and in pathological conditions (66). The 3G5 antigen, a ganglioside, is reported to be a useful marker for the identification of the corneal keratocytes and for documenting their response to stress associated with the wound healing (67). A previous study reported that FS-LASIK and SBK showed a little more severe keratocyte reaction compared to LASIK after 1–3 months of surgery due to the thinner corneal flap creation (17). The above observations indicate that the changes of GD3 27:4 levels might be associated with the wound healing after the refractive surgery.

An increased level of TAG 59:3 in week 52 was observed in the FS-LASIK and LASIK group compared to week 0, while there was no obvious change in the SBK group. Similarly, a clear separation between week 0 and 52 in the FS-LASIK and LASIK groups was observed, while the lipidomic profile of the SBK group in week 0 and 52 overlapped as shown in the PLS-DA score plot. No association was found between TAG 59:3 and the lipidomic profile of the SBK group in week 52. Chen et al. reported the upregulated TAGs in the meibum of the patients with dry eyes (68). Higher TAG has been demonstrated to be related to the corneal nerve damage in the patients with idiopathic small fiber neuropathy (69). In addition, it has been reported that the recovery rate of the corneal sub-basal nerve fibers in the SBK group was faster compared to the FS-LASIK and LASIK groups (17). The higher levels of TAG 59:3 observed at week 52 in the FS-LASIK and LASIK groups suggest that TAG 59:3 might play a negative role in the corneal sub-basal nerve fiber repairing.

(O-acyl)-ω-hydroxy fatty acid, an amphiphilic component in tears, plays a role in orienting the molecules at the lipid/water interface and facilitating the interaction between the polar and non-polar components of the tears to maintain the tear film stability (70). Lam et al. reported that the level of OAHFA was positively correlated with TBUT, reductions in ocular evaporation rate, and degree of ocular discomfort in the patients with dry eyes (3, 42). In this study, an obvious decrease of OAHFA intensity in week 52 was observed, while the intensity at week 0, week 1, and week 4 did not change much (Figure 4C). However, an increase of TBUT in week 52 in the SBK groups was detected (Table 2) showing a discrepancy with the previous studies (3, 42). The inconsistency between this study and Lam study might be due to the different cohorts of the patients. Their findings were based on a cohort of the patients with Meibomian gland dysfunction, while this study recruited only the patients with myopia.

The non-polar lipids, including CE, DAG, TAG, and WE, reside on the surface of an aqueous film and, thus, prevent the excessive evaporation of the aqueous component in the tear film. The deficiencies of the non-polar lipids may play a critical role in the evaporative dry eye (71). We did not detect any significant changes of the overall non-polar lipids preoperatively and postoperatively in the three types of refractive surgery (Figure 4). Only a significant alteration of DAG in the FS-LASIK group (Figure 5A) and WE in the SBK group (Figure 5C) was observed when examining the lipid species of an individual within the non-polar lipids. WEs are a major component of TFLL and recent studies have shown that WE film can effectively retard the evaporation of water (72, 73). Lam et al. reported a positive correlation between high molecular mass of the WEs with unsaturated FA chains and corneal staining, implying that the alteration of WEs level in the patients with dry eye syndrome was dependent on their molecular masses and fatty acyl chain saturation (9). However, different patterns were observed between the expression levels of WE and TBUT and Schirmer test in the SBK group. The assessment of overall levels of WE despite the fatty acyl saturations and molecular mass in this study might be a possible reason for this observation. Therefore, further evidence is required to determine the role of WE prior to and after the refractive surgery.

Phospholipids, accounting for 5–20 mol% of all the lipids in tears (74), play an essential role in the surface-active behavior of the meibum-like lipid compositions (75) and, thus, maintain the function of TFLL. This class of lipid could act as an interface between the Meibomian oil and the aqueous layer, since it lies anterior to the aqueous components. The presence of this polar phospholipid interface is critical to the spreading of the non-polar lipid film over the aqueous layer (76). Lysophospholipids were released by the hydrolysis of the phospholipid and this process was catalyzed by phospholipase A2. We have detected the significant changes in the phospholipids in the LASIK and SBK groups. Peters et al. found that TBUT was improved by the presence of the phospholipids by using a model eye (77). The trends of TBUT in the LASIK and SBK groups over time are similar compared to the PCs, which is a major component of the phospholipid. Those facts implicated that PC is responsible for the alterations of the overall phospholipids in the LASIK and SBK groups.

Our study findings must be considered in light of their limitations. First, a relatively small sample size was assessed in this study. Most clinical examination characteristics preoperatively and postoperatively did not achieve the statistical significance, most likely due to the small sample size. Furthermore, the inclusion of several time points between week 4 and 52 would provide more information for the lipidomic profile changes over time.

In this study, a rapid direct injection shotgun lipidomics workflow (<2 min/sample) was developed to examine the human tear lipidome from the patients receiving LASIK, FS-LASIK, or SBK surgery preoperatively and postoperatively (week 0, week 1, week 4, and week 52). The PLS-DA score plots revealed that the lipidome of the SBK group in week 52 was similar compared to week 0, while the FS-LASIK and LASIK groups showed distinct separation between week 0 and 52. Two lipids, TAG 59:3 and GD3 27:4, were found to be associated with the pattern changes among the FS-LASIK, LASIK, and SBK groups. LASIK showed the greatest impact on the lipidome changes over time. SBK demonstrated minimal impact among the three types of the refractive surgery after 1 year of surgery. Those findings in this longitudinal study could potentially aid in the understanding of the impact of the refractive surgery on the stability of the tear film by examining the human tear lipidome.

## Data Availability Statement

The original contributions presented in the study are included in the article/Supplementary Material, further inquiries can be directed to the corresponding author/s.

## Ethics Statement

The studies involving human participants were reviewed and approved by Ethics Committee of Tianjin Medical University Eye Hospital; Tianjin Medical University Eye Hospital. The patients/participants provided their written informed consent to participate in this study.

## Author Contributions

SZ and LZ designed the study and verify the underlying data. Preoperative patients were screened and surgeries were performed by SZ. YQ followed up the patients, collected tears from patients, and led the data collection. YG led the tear lipid layer analysis. YH and XL oversaw the research. YQ and YG wrote the first draft of the manuscript. All authors contributed to the article and approved the submitted version.

## Funding

This study was funded by the National Natural Science Foundation (NSFC #81970769, Beijing, China), the National Medical Research Council Centre (CG 2013 and CG 2017, Singapore), and the SingHealth Foundation (Singapore).

## Conflict of Interest

The authors declare that the research was conducted in the absence of any commercial or financial relationships that could be construed as a potential conflict of interest.

## Publisher's Note

All claims expressed in this article are solely those of the authors and do not necessarily represent those of their affiliated organizations, or those of the publisher, the editors and the reviewers. Any product that may be evaluated in this article, or claim that may be made by its manufacturer, is not guaranteed or endorsed by the publisher.
