# Supplementary material for: Lipidomics Analysis of the Tears in the Patients Receiving LASIK, FS-LASIK, or SBK Surgery
Source: Front Med (Lausanne). 2021 Oct 27;8:731462. doi: 10.3389/fmed.2021.731462 (PMC8579130; doi:10.3389/fmed.2021.731462)
Supplement: Supplementary file 1 [file Table_1.DOCX]

Supplementary Figures


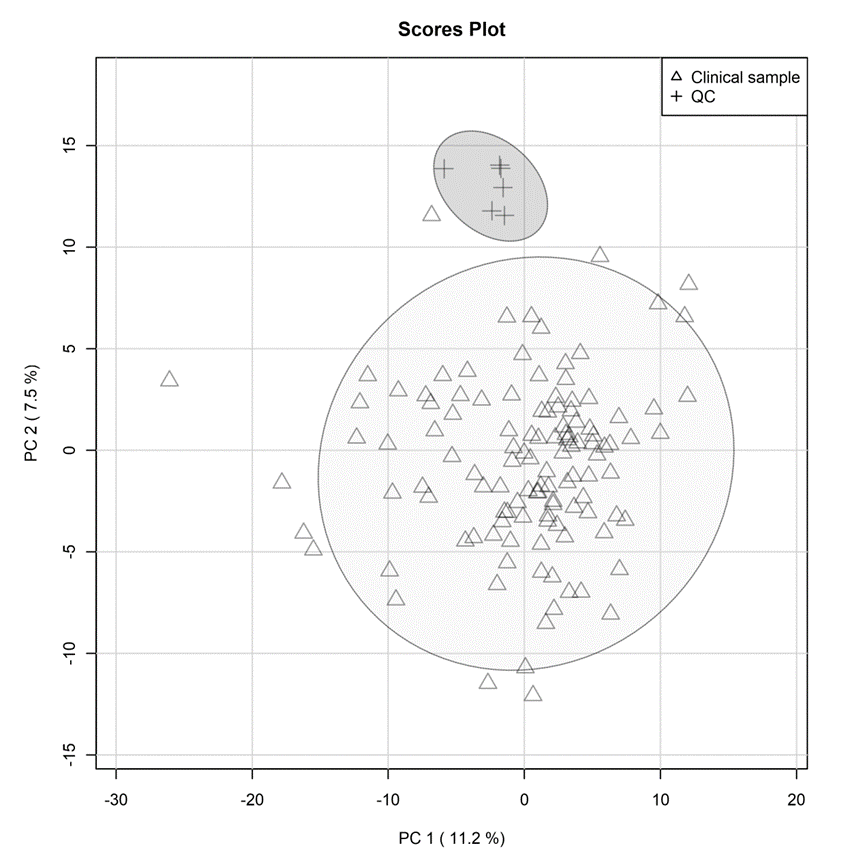


**Supplementary Figure 1.** PCA score plot showing clinical samples and quality control (QC).


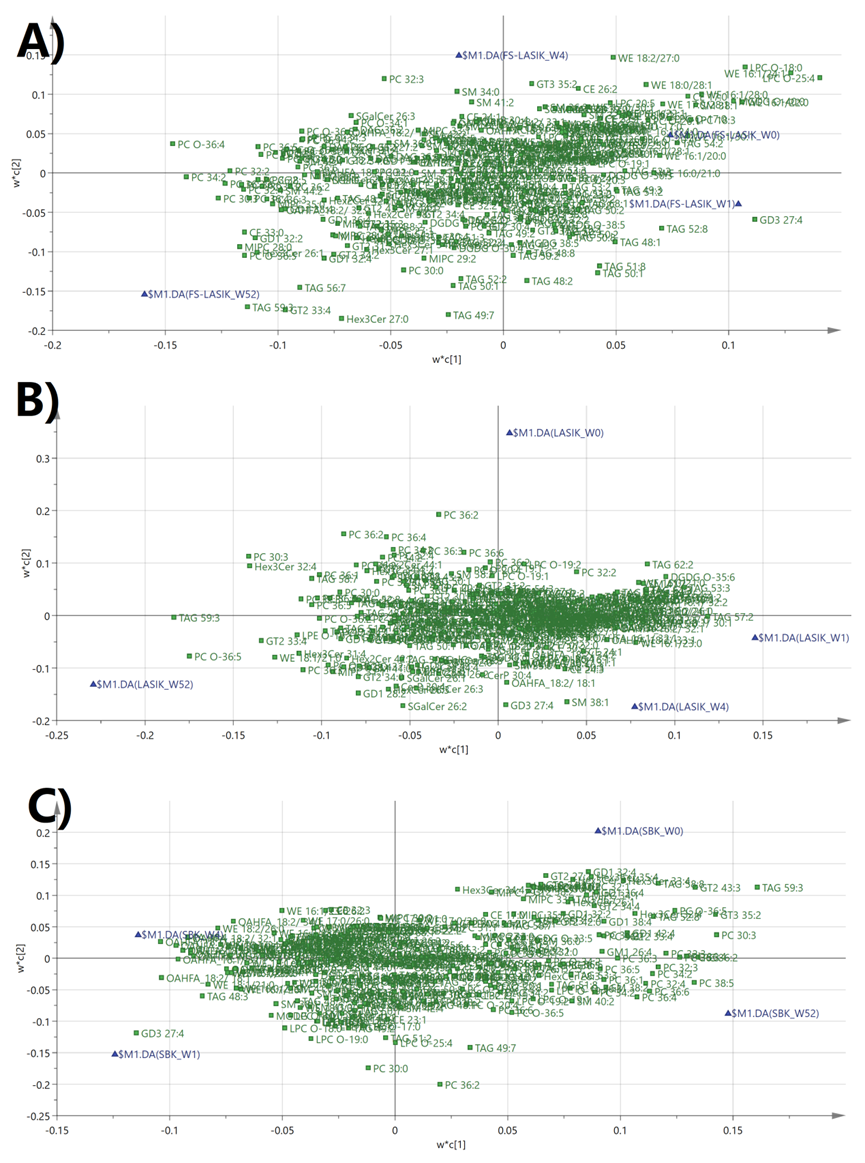


**Supplementary Figure 2.** PLS-DA loading plots. A) FS-LASIK; B) LASIK; C) SBK. Green square: X variables, i.e. lipid; Blue triangle: Y variables, i.e. time points.
